# *Aeschynomene*'s shortcut: A receptor-like cytoplasmic kinase drives nod-independent nodule formation

**DOI:** 10.1093/plcell/koaf202

**Published:** 2025-08-18

**Authors:** Min-Yao Jhu, Chai Hao Chiu

**Affiliations:** Assistant Features Editor, The Plant Cell, American Society of Plant Biologists; Crop Science Centre, Department of Plant Sciences, University of Cambridge, Cambridge CB3 0LE, UK; Crop Science Centre, Department of Plant Sciences, University of Cambridge, Cambridge CB3 0LE, UK

Legume crops can grow without added nitrogen fertilizers thanks to a remarkable partnership with rhizobia, soil bacteria that fix atmospheric nitrogen within specialized root nodules. In most legumes, nodule formation is initiated when rhizobia release Nod factors, small signaling molecules that plant receptors recognize to start symbiotic development. But some tropical legumes, such as *Aeschynomene evenia*, defy this rule: they form nitrogen-fixing nodules without recognizing Nod factors (Chaintreuil et al. 2018), partnering with photosynthetic *Bradyrhizobium* strains that live in flooded tropical soils. How these plants detect and accept their bacterial partners without the classical Nod factor signal has fascinated researchers for decades.

**Horta Araújo and colleagues (Horta Araújo et al. 2025)** tackled this question using the legume *Aeschynomene evenia*, a model species for Nod-independent nodulation. Through forward genetics, they screened ethyl methane sulfonate–mutagenized plants and identified mutants defective in nodule formation (Quilbé et al. 2021, 2022). In this recent work, they characterized mutants that hardly form nodules, forming on rare occasions 1 or a few enlarged nodules. Mapping-by-sequencing revealed the causal gene *AeRLCK2*, a Receptor-Like Cytoplasmic Kinase (RLCK). Genetic complementation via *Agrobacterium rhizogenes-*mediated hairy root transformation showed that restoring *AeRLCK2* fully rescued nodulation, confirming its essential role.

Using protein–protein interaction assays, subcellular localisation, co-immunoprecipitation, and phosphosite analysis, the authors showed that AeRLCK2 directly interacts with AeCRK, a Cysteine-Rich Receptor-Like Kinase previously shown to be required for Nod-independent symbiosis from the same forward mutant screen (Quilbé et al. 2021, 2022). AeCRK phosphorylates AeRLCK2 at specific serine/threonine (S/T) residues, and the functions were then further probed through mutagenesis and genetic complementation. Plants expressing the phospho-silent version of AeRLCK2 failed to fully recover nodulation, demonstrating that apart from the 5 S/T residues identified, post-translational modifications on other residues are also crucial for its symbiotic function.

Interestingly, despite *AeRLCK2* being expressed in both arbuscular mycorrhizal (AM) and nodulating roots, mutant analysis showed that *AeRLCK2* is dispensable for AM colonization in *A. evenia* (Fig. 1). Its closest paralog, *AeRLCK1*, shares sequence similarity but does not compensate for the loss of *AeRLCK2* during Nod-independent symbiosis, indicating functional divergence following a recent gene duplication event specific to Nod-independent *Aeschynomene*.

**Figure 1.**
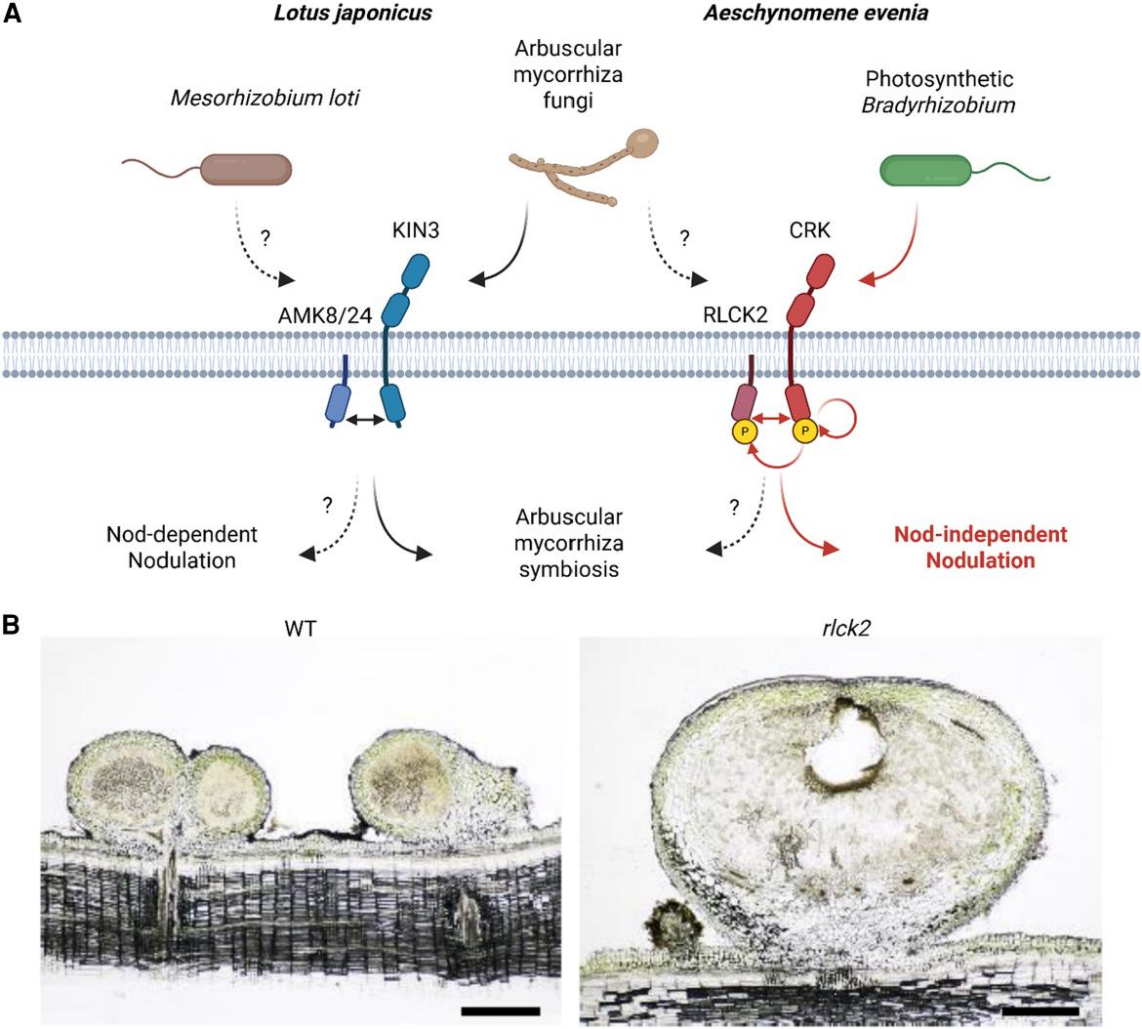
RLCK functions in AM and Nod-independent symbiosis in legumes, and nodulation phenotypes of *Aeschynomene evenia* mutants. **A)** In *Lotus japonicus*, the receptor-like cytoplasmic kinases AMK8 and AMK24 interact with the receptor-like kinase KIN3 at the periarbuscular membrane to coordinate AM symbiosis through reciprocal phosphorylation. Although *AMK8* and *AMK24* are also expressed during nodulation in *Lotus*, their exact role in rhizobial symbiosis remains unclear. In *Aeschynomene evenia*, the *AMK24*-like gene is absent, but 2 *AMK8*-related kinases, AeRLCK1 and AeRLCK2, are present. *AeRLCK2* is expressed during AM colonization but is not required for this process. Instead, AeRLCK2 is essential for Nod-independent nodulation with photosynthetic bradyrhizobia; it interacts with and is phosphorylated by the cysteine-rich receptor-like kinase AeCRK at the plasma membrane, triggering nodule formation. The bacterial signal activating this pathway and the downstream signaling steps remain unknown. Figure adapted from Figure 9 of Horta Araújo et al. (2025) and created with BioRender.com. **B)** Brightfield cross-sections of wild-type (WT) and *rlck2-11* mutant nodules at 21 days post-inoculation (dpi). WT roots produced an average of 35 nodules per root, whereas *rlck2* mutants typically formed only 1 or 2 BNs. Microscopy revealed that BNs had a similar internal structure to WT nodules, with central infected tissues surrounded by an uninfected cortex and vascular bundles, but often showed small necrotic zones and brown spots. Figure adapted from Figure 3D of Horta Araújo et al. (2025).

Comparative genomic analysis revealed how this specialization arose. In *Lotus japonicus*, the RLCKs AMK8 and AMK24 work with the receptor-like kinase KINASE3 (KIN3) to coordinate AM symbiosis at the periarbuscular membrane (Leng et al. 2023). This signaling module is likely conserved, with orthologous OsRLCK171 and OsARK1/ARK2 also functioning in arbuscular mycorrhiza symbiosis in rice (Roth et al. 2018; Montero et al. 2021; Leng et al. 2023). In *Lotus*, these kinases activate each other through phosphorylation, driving downstream signaling for arbuscule formation. *AMK8* and *AMK24* are also expressed during nodulation in *Lotus*, but their role in rhizobial symbiosis remains unresolved (Fig. 1), leaving open the question of whether similar dual-use modules exist in other legumes.

Horta Araújo et al. propose that *AeRLCK2* represents an example of neofunctionalization: an *AMK8*-like RLCK duplicated and evolved to mediate Nod-independent symbiosis with photosynthetic Bradyrhizobia. This innovation shows how legumes can modify conserved RLK/RLCK building blocks to adapt to new ecological niches and bacterial partners, highlighting the plasticity of plant–microbe signaling pathways.

Despite these new insights, key mysteries remain. The upstream bacterial signal that activates the AeCRK–AeRLCK2 complex has yet to be identified despite growing evidence suggesting that this complex is important for Nod-independent symbiosis. It is also unclear how this unique Nod-independent pathway links to the conserved common symbiosis signaling pathway, which coordinates the intracellular accommodation of microbes. Understanding these steps could help answer whether similar mechanisms exist in other Nod-independent legumes or actinorhizal plants.

Notably, when *AeRLCK2* is lost, mutant plants occasionally produce only a few Big Nodules (BN) instead of normal infection-filled nodules (Fig. 1B). Microscopy shows these BNs have organized internal tissue with an infected core and cortex but often develop small necrotic patches. This suggests nodule development can sometimes begin but fail to fully support rhizobial infection in the absence of AeRLCK2, resulting in enlarged but inefficient nodules. Therefore, understanding the stages and tissue contexts in which AeRLCK2- and AeCRK-mediated signaling operates will provide deeper insights into the initiation and maintenance of symbiosis.

Resolving these questions could reshape our understanding of how plants evolved diverse strategies to harness microbial nitrogen fixation, with exciting implications for engineering nitrogen-fixing traits in cereals and other non-legumes to support more sustainable agriculture.

## Recent related articles in *The Plant Cell*

- (Leng et al. 2023) showed that AMK8 and AMK24 associate with the receptor-like kinase KINASE3 to form an evolutionarily conserved RLK/RLCK complex that regulates arbuscular mycorrhizal symbiosis in *Lotus japonicus*.
- (Wang et al. 2022) reviewed how plants distinguish symbionts from pathogens and trace how overlapping and distinct signaling modules evolved to coordinate mycorrhizal and rhizobial symbioses.
- (Buscaill and van der Hoorn 2021) discussed how pathogens and symbionts deploy nine extracellular strategies to evade plant MAMP recognition, highlighting how plants distinguish beneficial partners from potential threats.

## Dive Curated Terms

The following phenotypic, genotypic, and functional terms are of significance to the work described in this paper:
